# Determination of miRNA expression profile in patients with prostate cancer and benign prostate hyperplasia

**DOI:** 10.55730/1300-0144.5374

**Published:** 2022-03-05

**Authors:** Okan SANCER, Pınar ASLAN KOŞAR, Muhammet Yusuf TEPEBAŞI, Osman ERGÜN, Murat DEMİR, Alim KOŞAR

**Affiliations:** 1 Department of Medical Biology, Faculty of Medicine, Süleyman Demirel University, Isparta, Turkey; 2 Department of Medical Genetic, Faculty of Medicine, Süleyman Demirel University, Isparta, Turkey; 3 Department of Urology, Faculty of Medicine, Süleyman Demirel University, Isparta, Turkey

**Keywords:** Benign prostate hyperplasia, microarray, miRNA, prostate cancer

## Abstract

**Background/aim:**

It is a known fact that the role of microRNAs (miRNA) has a very important place in cancer development and progression. miRNAs target a significant part of pathways as well as genes. This study aimed to compare the differential expression profiles of miRNAs in prostate cancer and benign prostatic hyperplasia patients.

**Materials and methods:**

Peripheral blood mononuclear cells (PBMCs) and tissue samples were collected from prostate cancer (PCa) (n: 20) and benign prostatic hyperplasia (BPH) (n: 20) patients. Total RNA isolation was performed. As a result of the RNA concentration and purity measurement, each patient group was pooled, and the miRNAs profiles comparison was performed with the Affymetrix Microarray System.

**Results:**

In tissue samples, 37 different expressed miRNAs were identified in PCa patients compared to BPH patients. In PBMCs samples, 27 different expressed miRNAs were identified in PCa patients compared to benign prostatic hyperplasia patients. As a result of the comparison of tissue and PBMCs samples, it was determined that down regülated hsa-miR-494-3p, hsa-miR-3128, hsa-miR-8084 were common miRNAs. 3 (HIF1A, NHS, INSL4) targets identified for hsa-miR-494-3p, 2 (HIF1A, AVRP1A) for hsa-miR-3128, 3 (AVRP1A, NHS, INSL4) for hsa-miR-8084.

**Conclusion:**

Our results suggested that determined common hsa-miR-494-3p, hsa-miR-3128, hsa-miR-8084 and their target HIF1A, AVRP1A, NHS, INSL4 may play a crucial role in therapeutic and early diagnostic strategies for prostate cancer. The present study may represent the first in-depth analysis of PBMCs and tissue samples miRNA profiles.

## 1. Introduction

PCa in men has been identified as the most diagnosed cancer type when examining the frequency of cancer types in terms of new cases and deaths in the World in 2018 [1]. Race differences, lifestyle, diet, age, family history, genetic polymorphisms, and socioeconomic causes are among the factors affecting the incidence of PCa. There are many reasons why the frequency of PCa varies in the world. Genetic and environmental factors constitute these two main elements [2,3].

In the early diagnosis of PCa, prostate-specific antigen (PSA) level [4], rectal examination, and transrectal ultrasonography prostate biopsy methods are used quite frequently [5,6]. Serum PSA is an important marker used to monitor potential PCa patients. In addition, PSA value appears to increase in prostate diseases such as BPH and prostatitis. PSA is an organ-specific but not cancer-specific test marker [6,7]. BPH, one of the prostate diseases, is a nonmalignant but proliferative disease. The volume of the prostate increases with age in normal men due to BPH. BPH is not cancer, and it is not usually a serious threat to health. If the prostate becomes enlarged, it can place pressure on the bladder and the urethra. As a result, difficulties in starting to void, frequent urination, and the inability to empty the bladder can be seen. Global incidence has in aged 50 and over more than 210 million men. Their pathogenesis is largely unknown [8–10].

Since early-stage solid tumors have higher growth potential than advanced-stage solid tumors, they undergo apoptosis and necrosis less frequently. As a result of this, early-stage solid tumors give restricted amounts of extracellular molecules into the circulation. The available circulating extracellular biomarkers generally show a low correctness for the early determination of solid tumors. Also, the molecular changes of PBMCs are not reliant on substantial tumor burden [11].

The rationale for using PBMCs as a biomarker for solid malignant tumors is based on the mechanism by which malignant growth causes characteristic changes in the blood biochemical environment. These changes are mostly related to the immune evasion mechanism of the tumor itself and will affect the expression pattern of some genes in blood cells [12]. Many miRNAs play a role in the regulation of the expression level of these genes.

In the immune system, peripheral blood mononuclear cells (PBMCs) act as the first line of defense against malignancy, but so many studies have identified the potential miRNA profiles for malignancies in a single sample, such as tissue or serum/plasma instead of PBMCs. However, in the same study; a) comparison of tissue samples and PBMCs samples with microarray system separately between PCa and BPH patient groups, b) determination of miRNAs that overlap in miRNA expression level in two different cell types, c) possible targets and pathways of these miRNAs determined in PCa patients research is almost nonexistent. The present study may represent the first in-depth analysis of PBMCs and tissue samples miRNA profiles.

## 2. Materials and methods

### 2.1. Patients and samples

From 40 patients who were followed up with clinical BPH and PCa diagnosis, 4 different groups were formed: BPH tissue (n: 20) and BPH PBMCs (n: 20), PCa tissue (n: 20), and PCa PBMCs (n: 20). Prostate tissue and whole blood samples were collected from Süleyman Demirel University Hospital (Isparta, Turkey) between January 2021 and August 2020.

The high dimensionality and complexity of the correlation structure in microarray data, however, there have been no sample size calculation methods accurately reflecting the true correlation structure of real microarray data [13]. Four to six samples per group have been described as adequate in microarray analysis [14].

Biopsy samples were taken by urology specialists from the area where the tumor tissue was located, under the guidance of ultrasonography. The biopsy materials were snap-frozen in liquid nitrogen after surgical resection and then stored at −80 °C with the RNALater (Qiagen, Germany) solution in cryotubes until RNA extraction. Fifty micrograms of each sample tissue were homogenized in Hibrigen HT buffer reagent with liquid nitrogen using an ultrasonication (Bandelin Sonopuls HD 2070, Bandelin Elec., Germany).

The patients who were diagnosed with PCa and BPH randomly selected. None of the patients had received any cancer therapy before the blood collection. It was determined as a result of the examinations made by the clinician that the patients did not have any malignancy except for any prostate cancer clinically.

### 2.2. PBMCs separation from the whole blood sample

Peripheral venous blood was taken into the K_2_ EDTA coated tubes (BD, Franklin Lakes, NJ). Within one hour of blood draw, PBMCs were isolated as described by Huang et al. [15]. PBMCs samples were stored at −80 °C until the total RNA extraction.

### 2.3. Total RNA Extraction from tissues and PBMCs

Total RNA isolation was performed by Hibrigen Total RNA extraction kit (MG-RNA-01-100) according to the manufacturer’s protocol. A total of 250 μL PBMCs and 50 mg homogenization of tissue samples were used.

### 2.4. Quality control of the total RNA and pooled samples

RNA samples concentration and purity were determined using NanoDropND-1000 spectrophotometer (NanoDrop Technologies, Inc., Wilmington, DE). 260/280 nm ratio was between 1.8 and 2.0. For four groups, the pooling of RNA samples has performed at equal concentrations. Final concentrations in the pools were verified again by NanoDrop spectrophotometry.

### 2.5. Microarray labeling/washing/staining/scanning procedure

The profiles of miRNAs was identified with the Affymetrix Microarray System. GeneChip miRNA 4.0 (Affymetrix, USA) commercial kit used in this system contains 30,434 mature miRNAs from 203 organisms and 2.025 pre-miRNA from humans, 2578 human mature miRNAs Release 20.0 miRBase set [16].

The following operations were performed sequentially with reference to the FlashTag Biotin HSR RNA Labeling Kit procedure. Total RNA (200 ng) was labeled with FlashTag Biotin HSR RNA Labeling Kit (Affymetrix, USA). Then, array chips were hybridized with a GeneChip Hybridization Control Kit (Affymetrix, USA). Hybridization conditions were at 60 rpm, 48 °C for 16 h. The chips arrays were washed and stained on a Fluidics Station 450 with AGCC Fluidics Control Software. Fluorescence was scanned with an Affymetrix GeneChip 3000-7G Scanner [16].

### 2.6. Statistical analysis

The following operations were performed sequentially with reference to the TAC 4.0.2 user manual. Files of raw data to be processed for analysis of miRNAs were created in ^*^CEL format with Affymetrix GeneChip Command Console Software. Normalization of the data obtained after image analysis was applied with the Transcriptome Analysis Console (TAC) Software (version 4.0.2.15) provided by ThermoFisher/Affymetrix. The signal intensities of the normalized sequences were compared with tests such as t-test, ANOVA, and significance analysis of the microarray after *log2* translation. At least 2-fold differences (increase or decrease) in microRNA expression at p < 0.05 were assumed to be significant [16]. Comparison of PSA and prostate value the two groups was conducted using the Mann–Whitney U test.

## 3. Results

### 3.1. Patient information

The age of patients ranges from 51 to 65 years. While the mean age in PCa is 63 ± 2.9, it is 62.25 ± 4 in BPH. The total PSA value mean was found to be 10.469 ± 11.6 ng/mL in the PCa patient group, 6.3705 ± 8 ng/mL in the BPH patient group, the prostate volume mean 67.75 ± 25 mL in the PCa patient group and 98.5 ± 42.8 mL in the BPH patient group. A statistical difference was found when the PSA and prostate volumes of PCa and BPH patients were compared (p < 0.05). PCa patients clinical information; TNM stage T1a (n: 4), T1b (n: 5), T2a (n: 5), T2b (n: 1), T2c (n: 2), T3b (n: 3), Gleason score 3+3 (n: 10), 3+4 (n: 2), 4+4 (n: 3), 4+5 (n: 1), 5+4 (n: 3), 5+5 (n: 1). All patients clinical information given in Table 1.

### 3.2. Array results

As a result of array study the miRNAs identified using TAC software are shown in Tables 2 and 3. The tables show the up/downregulated miRNAs of PCa group compared to BPH group in tissue and PBMCs samples. Table 2 includes 13 miRNAs that upregulate in the tissues and 24 miRNAs that downregulate. Table 3 includes 16 miRNAs that upregulate in the tissues and 11 miRNAs that downregulate.

### 3.3. Prediction of the targets of the common prognostic miRNAs

We predicted the targets of the three prognostic miRNAs using the TAC software and miRTargetLink Human online analysis tools. Each analysis tool identified unique targets for the three miRNAs, but all two tools identified 3 (HIF1A, NHS, INSL4) overlapping targets for hsa-miR-494-3p, 2 (HIF1A, AVRP1A) for hsa-miR-3128, 3 (AVRP1A, NHS, INSL4) for hsa-miR-8084 (Figure). These results suggest that overlapping miRNAs and their targets may play a role in prostate cancer development and progression.

## 4. Discussion

There are different methods such as tumor marker tests and imaging systems used in the diagnosis of PCa. In our study, we compared the results of the PSA test and prostate volume measurement used in the diagnostic methods of PCa and BPH patients. In the PCa group, the total PSA was higher than the BPH group, while the prostate volume was lower. We think the reason for the higher prostate volume in the BPH group, may be caused by hormonal changes that occur with increasing age.

However, we can explain the high level of total PSA in PCa patients despite the low prostate volume that with the expression that PSA release per unit volume is higher in the circulation in previous studies [17,18].

Although there are different methods such as tumor marker tests and imaging systems used in the diagnosis of PCa, understanding the molecular biology of cancer is very significant in the early diagnosis and accurate treatment of PCa.

miRNAs are used as a guide in the regulation of gene expression in many types of cancers, diagnosis, prognosis, and treatment alternatives [19]. Although plasma/serum miRNA research studies are a promising area for tumor markers for clinical applications, they lack sufficient sensitivity and specificity to improve early cancer detection [15]. PBMCs act as the first line of defense against malignancy in the immune system. In the case of dysfunctions, cancer immunogenicity or immune evasion may occur. Therefore, molecular profiles of PCMCs can provide new biomarkers for the early detection of malignancies. Importantly, molecular changes occurring in PBMCs are not dependent on tumor burden as in extracellular biomarkers [11]. We think that after the detection of biomarkers in tissues and PBMCs samples, the comparison of similarities between them is very important in the diagnosis/treatment of PCa and its differentiation from BPH.

Some recent researches have revealed that hsa-miR-494-3p, hsa-miR-3128, hsa-miR-8084 is involved in some human diseases, such as prostate cancer, colon cancer, breast cancer [20–22].

Previous studies have shown that hsa-miR-494-3p is closely associated with different types of cancer and has an increased expression level. It has been shown that this increase affects signaling pathway PI3K/AKT and its main target is PTEN [23–25]. On the other side, many studies including this one published by Li et al. [26] found that downregulation of microRNA-494 via loss of SMAD4 increases FOXM1 and β-catenin signaling in pancreatic ductal adenocarcinoma cells.

Overexpression of LINC01410 induced N-cadherin and vimentin expression while inhibiting E-cadherin expression in osteosarcoma cells. Overexpression of LINC01410 suppressed miR-3128 expression in MG-63 cells. These data indicated that LINC01410 acted as an oncogene [27]. Zhao et al. [28] found that LncRNA LOXL1-AS1 was higher expressed in nonsmall cell lung cancer tissues and promoted cancer progression by regulating miR-3128/RHOXF2.

Although not many studies on miR-8084 in the literature, Chong et al. [29] show that miR-8084 is the most significantly downexpressed in recurrent epithelial ovarian cancer.

In the present study, we found that the expression level of hsa-miR-494-3p, hsa-miR-3128, hsa-miR-8084 was significantly downregulated in prostate cancer tissues and PBMCs. These miRNAs predicted targets were HIF1A-NHS-INSL4, AVRP1A-HIF1A, AVRP1A-NHS-INSL4, respectively.

Tran et al. [30] found that hypoxia/HIF1a signaling pathways independently promote prostate cancer progression. Ivell et al. [31] expressed that nothing is known about roles for INSL4 in male reproduction and closely linked to the DMRT1 gene. INSL4 is not able to interact with RXFP1 and RXFP2 receptors. INSL4 is only known to be expressed in the human placenta, and is not detectable in any tissue of the male reproductive tract.

AVPR1A is expressed on various cell lines such as vascular smooth muscle, cardiomyocytes, hippocampus, kidney, bone, liver, and breast and nonsmall cell lung cancer. AVPR1A acts as receptor for arginine vasopressin. This receptor mediates cell contraction, proliferation, and glycogenolysis. Also, many available datasets from human castrate-resistant prostate cancer specimens show that AVPR1A gene copy number amplification [32]. AVPR1A inhibition had benefit for end stage bone-metastatic CRPC. Recent reports have defined to influence and tolerability of AVPR1A antagonism in autism spectrum disorder. Also, supports the use of again AVPR1A antagonists for advanced prostate cancer [33].

NHS protein is an actin regulatory protein that relevant role in cell morphology by maintaining the integrity of the circumferential actin ring and controlling lamellipodia formation [34,35].

hsa-miR-494-3p, hsa-miR-3128, and hsa-miR-8084 downregulated miRNAs in tissues and PBMCs may cause prostate cancer with changes in many points such as the regulations of the cell cycle, signal transduction, and cell communication. Also, it is mentioned in many studies that the predicted targets HIF1A, AVRP1A, NHS, INSL4 have a very important role in the progression of prostate cancer. Our findings in our study are not only effective miRNAs in prostate cancer but also related to other malignancies. Our aim is to reveal the effect of these miRNAs in prostate cancer.

In our study, miRNA expression levels are independent of the disease stage. It was tried to determine the relation of miRNAs with prostate cancer, not with metastasis. In our future studies, it is aimed to correlate the expression levels of these miRNAs with the progression of prostate cancer by looking at the relationship between TNM and Gleason scoring. Decreased levels of hsa-miR-494-3p, hsa-miR-3128, hsa-miR-8084 and therefore likely to increase levels of targets HIF1A, AVRP1A, NHS, INSL4 may be novel biomarker candidates for early-stage diagnosis and treatment of prostate cancer.

## Figures and Tables

**Figure:**
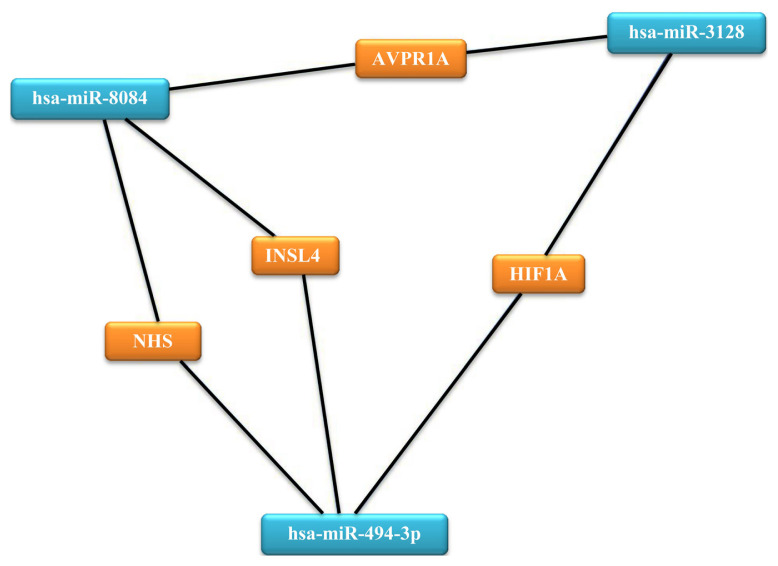
In PBMCs and tissue samples common miRNAs and their predicted targets.

**Table 1:** Clinical information of patients with PCa and BPH.

PCa					BPH		
Age	PSA value ng/mL	Prostate volume	TNM stage	Gleason score	Age	PSA value ng/mL	Prostate volume
65	6.51	90	T1b	3+3	64	16	115
65	5.02	80	T1a	3+3	65	9.7	130
62	4.74	70	T1a	3+3	65	4.78	80
65	1.9	50	T1b	3+3	65	3.14	150
65	8	50	T1a	3+3	51	3.59	90
65	18.94	75	T1a	3+3	65	4.49	80
60	8	50	T2b	4+4	62	3.23	60
60	16.24	60	T3b	4+4	64	3.05	75
65	13.37	100	T1b	5+4	65	37.22	200
64	10.94	150	T3b	4+5	65	6.01	180
65	1.18	50	T1b	5+4	60	1.69	40
65	10.53	50	T2c	3+4	65	1.58	60
56	6.6	60	T2a	3+3	64	5.04	100
58	4.8	45	T2a	3+3	65	2.2	60
60	6.23	75	T2a	3+3	65	4.44	80
60	4.91	55	T2a	3+3	58	1.77	120
65	56.15	70	T2c	5+4	55	1.77	120
65	5.14	45	T3b	5+5	65	9.4	75
65	9.18	50	T2a	3+4	59	3.96	45
65	11	80	T1b	4+4	58	4.35	110

**Table 2:** List of different expressed miRNAs of PCa group compared to BPH group in tissue samples.

Transcript cluster ID	Transcript ID (array design)	Accession number	Fold change (linear) (PCa vs. BPH)	Anova p-value (PCa vs. BPH)*	Chromosome
20503803	hsa-miR-494-3p	MIMAT0002816	−5.22	0.0296	chr14
20515528	hsa-miR-3128	MIMAT0014991	−4.06	0.029	chr2
20500723	hsa-miR-27b-3p	MIMAT0000419	−3.57	0.0308	chr9
20500162	hsa-miR-30a-5p	MIMAT0000087	−3.46	0.0142	chr6
20529794	hsa-miR-8084	MIMAT0031011	−3.26	0.0181	chr8
20500144	hsa-miR-22-3p	MIMAT0000077	−3.02	0.0037	chr17
20500181	hsa-miR-99a-5p	MIMAT0000097	−2.87	0.0004	chr21
20517675	hsa-miR-378c	MIMAT0016847	−2.86	0.0077	chr10
20525633	hsa-miR-6780b-5p	MIMAT0027572	−2.8	0.0043	chr6
20518834	hsa-miR-4454	MIMAT0018976	−2.76	0.0123	chr4
20504396	hsa-miR-642a-3p	MIMAT0020924	−2.69	0.0498	chr19
20500183	hsa-miR-100-5p	MIMAT0000098	−2.67	0.0016	chr11
20500148	hsa-miR-24-3p	MIMAT0000080	−2.66	0.0387	chr19
20501312	hsa-miR-345-5p	MIMAT0000772	−2.54	0.02	chr14
20524036	hsa-miR-6126	MIMAT0024599	−2.47	0.0056	chr16
20518930	hsa-miR-4529-3p	MIMAT0019068	−2.38	0.013	chr18
20501287	hsa-miR-151a-3p	MIMAT0000757	−2.37	0.0164	chr8
20500444	hsa-miR-181a-5p	MIMAT0000256	−2.32	0.0388	chr1
20500746	hsa-miR-140-3p	MIMAT0004597	−2.29	0.0189	chr16
20519554	hsa-miR-4721	MIMAT0019835	−2.26	0.0142	chr16
20501243	hsa-miR-378a-3p	MIMAT0000732	−2.25	0.0029	chr5
20500484	hsa-miR-221-3p	MIMAT0000278	−2.15	0.0106	chrX
20500752	hsa-miR-143-3p	MIMAT0000435	−2.14	0.0267	chr5
20500159	hsa-miR-28-3p	MIMAT0004502	−2	0.0439	chr3
20535911	hsa-miR-1282	MI0006429	2.06	0.0035	chr15
20525657	hsa-miR-6848-5p	MIMAT0027596	2.12	0.0033	chr8
20519405	hsa-miR-4632-5p	MIMAT0022977	2.22	0.0049	chr1
20501237	hsa-miR-375	MIMAT0000728	2.23	0.0092	chr2
20501293	hsa-miR-331-3p	MIMAT0000760	2.23	0.0138	chr12
20505790	hsa-miR-885-3p	MIMAT0004948	2.24	0.0391	chr3
20519609	hsa-miR-4750-5p	MIMAT0019887	2.57	0.0016	chr19
20506896	hsa-miR-664a-5p	MIMAT0005948	2.62	0.0064	chr1
20521786	hsa-miR-5572	MIMAT0022260	2.65	0.001	chr15
20515637	hsa-miR-3195	MIMAT0015079	2.69	0.0031	chr20
20523007	hsa-miR-6075	MIMAT0023700	2.85	0.0287	chr5
20518625	hsa-miR-3937	MIMAT0018352	3.34	0.0141	chrX
20517902	hsa-miR-3651	MIMAT0018071	3.57	0.0115	chr9

* The statistical significant level was set at p-value < 0.05. Greater than two fold changes were analyzed for down/upregulated miRNAs. In the table shown, a total of 37 miRNAs were found to be expressed differently.

**Table 3:** List of different expressed miRNAs of PCa group compared to BPH group in PBMCs samples.

Transcript cluster ID	Transcript ID (array design)	Accession number	Fold change (linear) (PCa vs. BPH)	Anova p-value (PCa vs. BPH)*	Chromosome
20503803	hsa-miR-494-3p	MIMAT0002816	−3.45	0.0002	chr14
20515528	hsa-miR-3128	MIMAT0014991	−3.01	0.0054	chr2
20502446	hsa-miR-451a	MIMAT0001631	−2.65	0.0004	chr17
20500128	hsa-miR-16-5p	MIMAT0000069	−2.52	0.0105	chr13
20520204	hsa-miR-5004-5p	MIMAT0021027	−2.45	0.0025	chr6
20529794	hsa-miR-8084	MIMAT0031011	−2.37	0.0175	chr8
20500121	hsa-let-7e-5p	MIMAT0000066	−2.34	0.0019	chr19
20500721	hsa-miR-23b-3p	MIMAT0000418	−2.33	0.0005	chr9
20515646	hsa-miR-3201	MIMAT0015086	−2.31	0.0008	chr22
20504554	hsa-miR-668-5p	MIMAT0026636	−2.27	0.022	chr14
20504316	hsa-miR-548a-3p	MIMAT0003251	−2.01	0.003	chr6
20500782	hsa-miR-150-5p	MIMAT0000451	2.04	0.0193	chr19
20518788	hsa-miR-378f	MIMAT0018932	2.06	0.0148	chr1
20500771	hsa-miR-127-3p	MIMAT0000446	2.07	0.014	chr14
20509237	hsa-miR-1915-3p	MIMAT0007892	2.14	0.0005	chr10
20537261	hsa-mir-6089-1	MI0020366	2.15	0.0138	chrX
20537862	hsa-mir-6089-2	MI0023563	2.15	0.0138	chrY
20525699	hsa-miR-6869-5p	MIMAT0027638	2.18	0.0061	chr20
20525553	hsa-miR-6796-5p	MIMAT0027492	2.23	0.0069	chr19
20518880	hsa-miR-4486	MIMAT0019020	2.23	0.0008	chr11
20519573	hsa-miR-4730	MIMAT0019852	2.26	0.0058	chr17
20500761	hsa-miR-191-5p	MIMAT0000440	2.35	0.0006	chr3
20519493	hsa-miR-4687-3p	MIMAT0019775	2.37	0.0288	chr11
20514163	hsa-miR-2861	MIMAT0013802	2.45	0.0134	chr9
20518815	hsa-miR-4440	MIMAT0018958	2.48	0.0115	chr2
20517729	hsa-miR-4270	MIMAT0016900	2.88	0.0028	chr3
20525561	hsa-miR-6800-5p	MIMAT0027500	4.22	0.0004	chr19

* The statistical significant level was set at p-value < 0.05. Greater than two fold changes were analyzed for down/upregulated miRNAs. In the table shown, a total of 27 miRNAs were found to be expressed differently.
